# Air pollution exposure associated with decline rates in skeletal muscle mass and grip strength and increase rate in body fat in elderly: a 5-year follow-up study

**DOI:** 10.1265/ehpm.24-00357

**Published:** 2025-07-18

**Authors:** Chi-Hsien Chen, Li-Ying Huang, Kang-Yun Lee, Chih-Da Wu, Shih-Chun Pan, Yue Leon Guo

**Affiliations:** 1 Department of Environmental and Occupational Medicine, National Taiwan University College of Medicine and National Taiwan University Hospital, Taipei, Taiwan; 2 School of Medicine, College of Medicine, Fu Jen Catholic University, New Taipei City, Taiwan; 3 Division of Endocrinology and Metabolism, Department of Internal Medicine, and Department of Medical Education, Fu Jen Catholic University Hospital, New Taipei City, Taiwan; 4 Division of Pulmonary Medicine, Department of Internal Medicine, Shuang Ho Hospital, Taipei Medical University, New Taipei City, Taiwan; 5 Department of Internal Medicine, School of Medicine, College of Medicine, Taipei Medical University, Taipei, Taiwan; 6 Department of Geomatics, National Cheng Kung University, Tainan, Taiwan; 7 National Institute of Environmental Health Sciences, National Health Research Institutes, Miaoli, Taiwan; 8 Institute of Environmental and Occupational Health Sciences, National Taiwan University College of Public Health, Taipei 100, Taiwan

**Keywords:** Air pollution, Particulate matter, Grip strength, Skeletal muscle mass, Body fat mass, Elderly

## Abstract

**Background:**

The effect of air pollution on annual change rates in grip strength and body composition in the elderly is unknown.

**Objectives:**

This study evaluated the effects of long-term exposure to ambient air pollution on change rates of grip strength and body composition in the elderly.

**Methods:**

In the period 2016–2020, grip strength and body composition were assessed and measured 1–2 times per year in 395 elderly participants living in the Taipei basin. Exposure to ambient fine particulate matters (PM_2.5_), nitric dioxide (NO_2_), and ozone (O_3_) from 2015 to 2019 was estimated using a hybrid Kriging/Land-use regression model. In addition, long-term exposure to carbon monoxide (CO) was estimated using an ordinary Kriging approach. Associations between air pollution exposures and annual changes in health outcomes were analyzed using linear mixed-effects models.

**Results:**

An inter-quartile range (4.1 µg/m^3^) increase in long-term exposure to PM_2.5_ was associated with a faster decline rate in grip strength (−0.16 kg per year) and skeletal muscle mass (−0.14 kg per year), but an increase in body fat mass (0.21 kg per year). The effect of PM_2.5_ remained robust after adjustment for NO_2_, O_3_ and CO exposure. In subgroup analysis, the PM_2.5_-related decline rate in grip strength was greater in participants with older age (>70 years) or higher protein intake, whereas in skeletal muscle mass, the decline rate was more pronounced in participants having a lower frequency of moderate or strenuous exercise. The PM_2.5_-related increase rate in body fat mass was higher in participants having a lower frequency of strenuous exercise or soybean intake.

**Conclusions:**

Among the elderly, long-term exposure to ambient PM_2.5_ is associated with a faster decline in grip strength and skeletal muscle mass, and an increase in body fat mass. Susceptibility to PM_2.5_ may be influenced by age, physical activity, and dietary protein intake; however, these modifying effects vary across different health outcomes, and further research is needed to clarify their mechanisms and consistency.

**Supplementary information:**

The online version contains supplementary material available at https://doi.org/10.1265/ehpm.24-00357.

## Introduction

In 2017, exposure to ambient particulate matter with an aerodynamic diameter <2.5 µm (PM_2.5_) was associated with 2.94 million deaths and 5.25% of all deaths worldwide, making it the eighth leading risk for mortality [1]. This represented a 6.7% increase compared to the 1.75 million deaths attributable to PM_2.5_ in 1990 [1]. The elderly is considered a susceptible population due to their lower organ reserve, higher prevalence of co-morbidities like cardiovascular, pulmonary, and metabolic diseases, and poorer anti-oxidative potential [2, 3].

Age-related decline in muscle strength and skeletal muscle mass is an inevitable process after mid-life [4]. Longitudinal studies show that people around the age of 75 years have an annual decline rate in muscle mass of 0.80–0.98% in men and 0.64–0.70% in women. The annual decline rate in muscle strength is likewise faster in men than in women (3–4% vs. 2.5–3%) [5].

Sarcopenia is a clinical condition of accelerated loss of muscle mass and function in the elderly. It is linked to several adverse outcomes, including falls, frailty, and mortality [6–8]. In a meta-analysis of prospective cohort studies, grip strength is an independent predictor of all-cause mortality and cardiovascular disease [9]. When sarcopenia is accompanied by increased body fat mass, known as sarcopenic obesity, the mortality risk becomes higher compared to each condition separately [10]. As such, muscle strength and body composition measurements may provide a useful assessment on the general health of the elderly.

The effects of long-term PM_2.5_ exposure on the annual change rates of grip strength and body composition remain unknown. One previous cross-sectional study has found that PM_2.5_ exposure is linked to lower skeletal muscle mass and higher body fat mass [11]. A recent large cross-sectional study reveals that in people aged >50 years, PM_2.5_ exposure is negatively associated with grip strength [12]. Previous understanding of the pathologic pathways of sarcopenia and the toxicologic mechanisms of PM_2.5_ may provide biological possibilities for the observed correlations. Major factors involved in the development and progression of sarcopenia include lifestyle factors (e.g. low physical activity, obesity, and smoking), malnutrition (e.g. low protein or calorie intake, vitamin D deficiency), chronic inflammation, decline in neural function, mitochondria dysfunction, hormonal changes (e.g. reductions in growth hormone, insulin-like growth factor 1, testosterone, and estrogens), and reduced satellite cell function [13].

Some pathologic pathways of sarcopenia overlap with PM_2.5_ induced toxicities. Epidemiologic studies reveal that PM_2.5_ exposure can increase systemic inflammatory biomarkers, including CRP [14–17], IL-6 [18], TNF-α [18], and white blood cell count [17]. Many animal and human studies provide evidence for the effects of PM_2.5_ on brain inflammation and neurodegenerative diseases [19]. *In vitro* [20] and animal studies [21, 22] reveal that PM_2.5_ causes mitochondrial dysfunction and damage, resulting in increased oxidative stress and lower cellular energy supply. Moreover, PM_2.5_ exposures have been negatively associated with blood testosterone levels in mice [23] and infertile men [24].

In addition, PM_2.5_ can increase vascular insulin resistance [25], obesity [26], and diabetes mellitus type 2 [26]. These are precursors and phenotypes of insulin resistance [27], which causes muscle fiber atrophy, lipid accumulation in muscles, and mitochondrial dysfunction. These, in turn, enhance muscle wasting, dysfunction, and oxidative stress [28]. Given the broad range of physiological effects induced by air pollution, including systemic inflammation, mitochondrial dysfunction, and hormonal alterations, selecting health outcomes that reflect these integrated pathophysiological processes is essential. Grip strength and body composition (skeletal muscle mass and body fat mass) serve as practical and clinically meaningful endpoints that capture the downstream consequences of these mechanisms. Grip strength is a well-established surrogate for neuromuscular function and overall vitality in older adults and is independently associated with adverse outcomes such as disability, hospitalization, and mortality [5, 8, 9]. Similarly, changes in muscle and fat mass reflect chronic metabolic and inflammatory disturbances [13, 28], as well as age-related anabolic resistance, all of which may be exacerbated by pollution-induced oxidative stress [3, 18, 20, 21] and endocrine disruption [23, 24]. Therefore, these outcomes provide a relevant and sensitive lens to examine how environmental exposures influence aging-related functional decline. This prospective cohort study on the elderly was therefore undertaken to examine the causal effects of ambient PM_2.5_ exposure on the annual change rates in grip strength, skeletal muscle mass, and body fat mass.

## Materials and methods

### Subjects

Between October 2015 and November 2016, a cross-sectional study was conducted among individuals aged 65 years and older residing in the Taipei Basin, Taiwan. Participants were recruited during their routine annual health examinations at two hospitals located in Taipei City and New Taipei City. Individuals were excluded if they had a malignancy, experienced significant ambulation difficulties that prevented independent walking or standing during assessments, or had severe communication impairments that interfered with understanding instructions or providing informed consent. From 2016 to 2020, participants returned 1–2 times a year to undertake examinations and assessment and measurements of handgrip strength and body composition on each visit. Sample size estimation was performed using G*Power (version 3.1.9.7) for repeated measures ANOVA with within-subject factors. Based on the effect size (f = 0.12) derived from our previous cross-sectional study of PM_2.5_-associated changes in body composition (standardized d ≈ 0.087) [11], five repeated measurements, α = 0.05, 80% power, and a conservative nonsphericity correction (ε = 0.6), the required sample size was estimated at approximately 120 participants. However, to account for potential attrition, missing data, and the need for subgroup analyses, a larger sample of 395 participants was followed over time, providing robust power for detecting associations between PM_2.5_ exposure and changes in body composition and grip strength. The institutional review board of the National Health Research Institutes (EC1040508-E-R2) approved this study and all of the study participants provided written informed consent.

### Questionnaire evaluation

Two well-trained research assistants conducted face-to-face interviews with the participants to facilitate the questionnaire answers. Physician-diagnosed medical conditions (e.g. diabetes mellitus, stroke, heart diseases, asthma, COPD, renal disease, and arthritis), educational attainment, smoking habit, indoor air pollution exposures (e.g. second-hand smoke, cooking, and incense burning), physical activity, and dietary frequency were assessed.

The frequencies of exposures to second-hand smoke, cooking (frying or stir-frying), and incense at home were collected through the questionnaire. For the first two conditions, an additional question regarding the time of each exposure (in minutes) was included. Monthly exposures to second-hand smoke and cooking oil fume were estimated by multiplying exposure frequency by time. Incense at home was divided into four levels: no contact; <1/week; 1–3/week; and >3/week.

Physical activity was assessed using the Chinese Edition of the Physical Activity Scale for the Elderly (PASE) questionnaire, which included 12 types of activities (i.e. walk outside home, light sports or recreational activities, moderate sports or recreational activities, strenuous sports or recreational activities, muscle strengthening, light housework, heavy housework, home repairs, lawn work, outdoor gardening, caring for another person, and work for pay or as a volunteer) [29, 30]. Light sports or recreational activities, such as stretching, fishing, singing, or playing a musical instrument, do not exert effort, sweat, or significantly increase breathing and heartbeat. Moderate sports or recreational activities, such as walking, tai chi, yuan chih dancing, folk dancing, and swimming or cycling at personal usual speed, can be a little strenuous, sweat a little, or increase breathing and heart rate slightly. Strenuous sports or recreational activities, such as running, climbing, playing ball, climbing stairs, aerobic dancing, fast swimming, or fast cycling, can be very strenuous, with rapid breathing, heartbeat, and profuse sweating. Each PASE item was assigned a weight based on energy expenditure (metabolic equivalent of the task, MET). The PASE score was the product of the activity weight multiplied by the frequency of each item [30].

The protein intake of seven categories was assessed through a series of dietary frequency questions, including fish/seafood (three items), poultry (one item), meat (four items), soybean and its products (two items), milk (three items), and eggs (one item). The frequency of each item was scored as 0 (never or less than once per month), 1 (1–3 times/month), 2 (1–2 times/week), 3 (3–4 times/week), 4 (5–6 times/week), 5 (1/day), 6 (2/day), and 7 (>3/day). Protein score was the aggregate scores of these seven categories.

### Handgrip strength measurement

Handgrip strength was measured using a dynamometer (TTM-YD, Tokyo, Japan) in a sitting position with the participant’s shoulders adducted, elbows flexed at 90°, and wrist in a neutral position. The participants performed three trials on each hand alternately. To obtain the best reliability and validity [31], the averages of the three tests for the left and right sides were calculated. The higher value was used for data analysis.

### Body composition measurement

Body composition was measured using bioelectric impedance analysis devices (BIA, Inbody 120, InBody Co., Ltd. Seoul, Korea) with the participant in a standing posture and lightly dressed. To minimize the influence of food and fluid intake, all of the participants underwent the examination in the morning, after at least 8 hours of fasting. Body height and weight were also measured. Parameters of body composition used for data analysis included skeletal muscle mass (SMM; in kg) and body fat mass (BFM; in kg).

### Air pollution exposure assessment

The monthly concentrations of PM_2.5_, NO_2_, O_3_, and CO were estimated using spatial-temporal modeling based on hourly measured data from 73 EPA monitoring stations in Taiwan. The model combined the Kriging model and land-use regression model, and had cross-validated R^2^ values of 0.88, 0.87, 0.80 for PM_2.5_, NO_2_, and O_3_, respectively [32, 33]. For carbon monoxide (CO), long-term exposure was estimated using a modified ordinary Kriging approach [34], with a cross-validated R^2^ value of 0.28. The residential address of each participant was geocoded to match the estimated concentration of air pollution. Long-term exposure to air pollution was the average of levels from 2015 to 2019.

### Statistical analysis

To account for repeated measurements and varying numbers of observations per participant, we used linear mixed-effects models to examine the association between long-term air pollution exposure and changes in grip strength and body composition over time. Time (in years since baseline) was treated as a continuous variable, and an interaction term between time and pollutant concentration was included to estimate the exposure-related annual change rate in each outcome. This interaction term reflects the additional yearly change in the outcome per interquartile range (IQR) increase in air pollution exposure. Models included random intercepts to account for between-subject variability and were adjusted for fixed covariates: sex, age at baseline, body height and weight, educational attainment, current and former smoking status, hypertension, diabetes mellitus, stroke, heart disease, asthma, chronic obstructive pulmonary disease, renal disease, arthritis, physical activity score, protein intake score, second-hand smoke exposure, cooking frequency, incense burning, and average ambient temperature and relative humidity in 2019. Time-varying covariates included time from baseline and season of testing. Season of testing was categorized as cold (November, December, February, March, and April) or warm (May through October), based on average regional temperature patterns.

Both single-pollutant and two-pollutant models were fitted to identify the pollutant most consistently associated with longitudinal changes in musculoskeletal health. To account for multiple comparisons, we applied false discovery rate (FDR) adjustment to the p-values using the Benjamini-Hochberg procedure. Associations with FDR-adjusted p-values < 0.05 were considered statistically significant and were further evaluated using two-pollutant models that included adjustments for co-pollutants.

The effect modifications of age, sex, chronic diseases, physical activities, and protein intakes were further examined. Stratified analysis was done to demonstrate the different effects of age (<70 years and ≥70 years), sex (male and female), hypertension (yes and no), diabetes mellitus (yes and no), heart diseases (yes and no), arthritis (yes and no), PASE score (<median and ≥median), light sports scores (<median and ≥median), moderate sports scores (<median and ≥median), strenuous sports scores (<third quartile and ≥third quartile), protein scores (<median and ≥median), animal meat protein scores (<median and ≥median), soybean product (<median and ≥median), milk (<median and ≥median), eggs (<median and ≥median), and season of testing (cold and warm). The animal meat protein scores were the summation of fish/seafood, poultry, and meat scores. Models with interaction terms were also conducted to examine the statistical significance of effect modification.

To further evaluate the potential modifying effect of seasonality, we conducted an additional subgroup analysis restricted to participants (n = 135) who had repeated measurements in both cold and warm seasons across different years, enabling within-subject comparisons of air pollution effects on the annual change rate of health outcomes under varying seasonal conditions.

All statistical analyses were done using the JMP pro 17 (SAS Institute Inc., USA). Statistical significance was set at *p* < 0.05.

## Results

A total of 395 elderly participants completed 1,945 visits between 2016 and 2020, with a mean follow-up duration of 4.1 ± 0.3 years (range: 3.0–4.6 years). Table 1 presents the baseline characteristics of the participants, stratified by tertiles of ambient PM_2.5_ levels at their residences in 2015. As shown in Supplementary Table 1S, most participants remained in the same PM_2.5_ exposure category during the follow-up period from 2015 to 2019, indicating stable long-term exposure levels across tertiles. The mean age of the cohort was 70.3 ± 3.9 years, and 41.3% were male. The average body mass index (BMI) was 24.3 ± 3.2 kg/m^2^, with 1.8% of participants underweight (BMI < 18.5) and 16.8% classified as obese (BMI ≥ 27). Approximately 35% of the participants had an education level lower than high school. At baseline, none of the participants met the diagnostic criteria for sarcopenia defined by the Asian Working Group for Sarcopenia. At baseline, participants residing in areas with higher ambient PM_2.5_ levels had significantly greater total body fat mass (p = 0.009) and fat mass in the arm (p = 0.002) and leg (p < 0.0001) regions. However, baseline grip strength (p = 0.633) and total skeletal muscle mass (p = 0.672) did not differ significantly across PM_2.5_ exposure groups. Despite the absence of statistically significant differences in muscle mass and grip strength at baseline, the follow-up data revealed that participants in the high PM_2.5_ group experienced the most pronounced annual increases in body fat mass (+0.10 ± 0.72 kg/year) and decreases in skeletal muscle mass (−0.13 ± 0.28 kg/year). These findings suggest that even in a population with initially comparable muscle mass and grip strength, higher long-term PM_2.5_ exposure was associated with an accelerated decline in musculoskeletal health over time.

**Table 1:** Characteristics of the 395 elderly participating in 1945 visits in this study, 2016–2020.

		**Ambient PM_2.5_ levels in 2015**			***P* value**
	**Total**	**Low** **(24.3 µg/m^3^)^a^**	**Medium** **(26.2 µg/m^3^)^a^**	**High** **(31.4 µg/m^3^)^a^**	
	**n = 395**	**n = 132**	**n = 132**	**n = 131**	
Baseline characteristics					
Age, mean ± SD, yr	70.3 ± 3.9	69.9 ± 3.8	70.4 ± 4.0	70.7 ± 3.8	0.315
Male, n (%)	163 (41.3)	54 (40.9)	50 (37.9)	59 (45.0)	0.496
Body mass index, mean ± SD	24.3 ± 3.2	24.2 ± 2.9	24.6 ± 3.5	24.1 ± 3.1	0.441
Education <13 yr, n (%)	137 (34.7)	45 (34.1)	55 (41.7)	37 (28.2)	0.072
Smoking status, n (%)					0.207
Never	340 (86.1)	112 (84.8)	121 (91.7)	107 (81.7)	
Former	47 (11.9)	17 (12.9)	9 (6.8)	21 (16.0)	
Current	8 (2.0)	3 (2.3)	2 (1.5)	3 (2.3)	
SHS exposure, min per month, median (IQR)	6 (6)	6 (0)	6 (6)	1 (19)	0.058
Cooking (frying), min per month, median (IQR)	300 (900)	120 (570)	380 (900)	600 (1800)	<0.0001
Incense burning at home, n (%)					0.009
Never	219 (55.4)	59 (44.7)	76 (57.6)	84 (64.1)	
<once a week	17 (4.3)	7 (5.3)	7 (5.3)	3 (2.3)	
once to trice a week	55 (13.9)	19 (14.4)	14 (10.6)	22 (16.8)	
>trice a week	104 (26.3)	47 (35.6)	35 (26.5)	22 (16.8)	
Hypertension, n (%)	151 (38.2)	54 (40.9)	50 (37.9)	47 (35.9)	0.699
Diabetes mellitus, n (%)	66 (16.7)	22 (16.7)	20 (15.2)	24 (18.3)	0.789
Stroke, n (%)	21 (5.3)	8 (6.1)	8 (6.1)	5 (3.8)	0.645
Heart disease, n (%)	59 (14.9)	16 (12.1)	23 (17.4)	20 (15.3)	0.478
Asthma, n (%)	9 (2.3)	1 (0.8)	3 (2.3)	5 (3.8)	0.251
COPD, n (%)	18 (4.6)	4 (3.0)	8 (6.1)	6 (4.6)	0.498
Renal disease, n (%)	21 (5.3)	8 (6.1)	8 (6.1)	5 (3.8)	0.645
Arthritis, n (%)	66 (16.7)	18 (13.6)	22 (16.7)	26 (19.9)	0.402
PASE score, median (IQR)	111.8 (83.8)	114.9 (78.2)	115.3 (89.9)	103.6 (85.7)	0.731
Light sports score, median (IQR)	9.0 (27.1)	9.0 (27.1)	9.0 (27.1)	9.0 (24.8)	0.849
Moderate sports score, median (IQR)	2.5 (17.3)	0 (29.7)	1.3 (28.4)	2.5 (17.3)	0.696
Strenuous sports score, median (IQR)	0 (9.9)	0 (9.9)	0 (9.9)	0 (9.9)	0.311
Score for protein dietary frequency, median (IQR)	22 (7)	22 (8)	22 (8)	22 (7)	0.338
Score for animal meat, median (IQR)	8 (3)	8 (4)	7 (3)	8 (3)	0.092
Score for soybean, median (IQR)	4 (2)	4 (3)	4 (3)	4 (3)	0.757
Score for milk, median (IQR)	2 (5)	2 (5)	2 (5)	3 (5)	0.728
Score for eggs, median (IQR)	3 (2)	3 (3)	3 (2.8)	3 (2)	0.054
Body fat mass, mean ± SD, kg	19.8 ± 5.9	18.5 ± 5.2	20.6 ± 6.4	20.2 ± 5.9	0.009
Skeletal muscle mass, mean ± SD, kg	22.5 ± 4.6	22.8 ± 4.5	22.3 ± 4.3	22.5 ± 4.9	0.672
Grip strength, mean ± SD, kg	25.6 ± 8.5	25.1 ± 8.5	25.6 ± 8.6	26.1 ± 8.5	0.633
Fat mass of arm, mean ± SD, kg	1.4 ± 0.6	1.2 ± 0.5	1.5 ± 0.6	1.4 ± 0.5	0.002
Fat mass of trunk, mean ± SD, kg	9.9 ± 3.1	9.4 ± 2.9	10.2 ± 3.3	10.0 ± 3.1	0.093
Fat mass of leg, mean ± SD, kg	3.0 ± 0.9	2.8 ± 0.7	3.2 ± 1	3.2 ± 0.9	<0.0001
Skeletal muscle mass of arm, mean ± SD, kg	2.1 ± 0.6	2.2 ± 0.6	2.0 ± 0.6	2.0 ± 0.6	0.046
Skeletal muscle mass of trunk, mean ± SD, kg	18.4 ± 3.6	18.9 ± 3.7	18.2 ± 3.5	18.1 ± 3.8	0.147
Skeletal muscle mass of leg, mean ± SD, kg	6.2 ± 1.4	6.2 ± 1.4	6.2 ± 1.4	6.3 ± 1.5	0.624
Follow-up duration, mean ± SD, yr	4.1 ± 0.3	4.1 ± 0.3	4.1 ± 0.3	4.2 ± 0.3	0.002
Annual change rate of body composition and grip strength^b^					
Body fat mass, mean ± SD, kg/year	−0.12 ± 0.82	−0.24 ± 0.88	−0.23 ± 0.8	0.10 ± 0.72	0.0005
Skeletal muscle mass, mean ± SD, kg/year	0.01 ± 0.36	0.13 ± 0.37	0.05 ± 0.37	−0.13 ± 0.28	<0.0001
Grip strength, mean ± SD, kg/year	−0.002 ± 0.88	0.14 ± 0.81	−0.03 ± 0.85	−0.11 ± 0.96	0.074
Fat mass of arm, mean ± SD, kg/year	−0.01 ± 0.08	−0.02 ± 0.09	−0.02 ± 0.08	0.02 ± 0.07	<0.0001
Fat mass of trunk, mean ± SD, kg/year	−0.04 ± 0.41	−0.07 ± 0.44	−0.07 ± 0.40	0.01 ± 0.38	0.190
Fat mass of leg, mean ± SD, kg/year	−0.03 ± 0.14	−0.07 ± 0.14	−0.06 ± 0.14	0.03 ± 0.12	<0.0001
Skeletal muscle mass of arm, mean ± SD, kg/year	0.005 ± 0.07	0.04 ± 0.06	0.01 ± 0.07	−0.03 ± 0.05	<0.0001
Skeletal muscle mass of trunk, mean ± SD, kg/year	0.002 ± 0.38	0.17 ± 0.34	0.05 ± 0.40	−0.21 ± 0.28	<0.0001
Skeletal muscle mass of leg, mean ± SD, kg/year	−0.02 ± 0.10	−0.01 ± 0.11	−0.02 ± 0.10	−0.03 ± 0.08	0.212

Definition of abbreviation: SHS = second-hand smoke; SD = standard deviation; COPD = chronic obstructive pulmonary disease; PASE = Physical Activity Scale for the Elderly.

^a^: mean concentration

^b^: The annual change rate of grip strength and body composition indices was calculated by linear regression for each participant.

Notably, participants exposed to higher PM_2.5_ levels were more likely to engage in frying or stir-frying at home (p < 0.001) and less likely to burn incense (p = 0.009), while other characteristics such as age, sex, chronic disease status, physical activity, and dietary scores showed no statistically significant differences across PM_2.5_ exposure categories. To account for the potential confounding effects of indoor air pollution sources on the observed associations with ambient PM_2.5_, we included duration of frying or stir-frying and incense burning frequency as covariates in all regression models.

The average exposure concentration of PM_2.5_ was 23.5 µg/m^3^, indicating that it is still an important air pollutant in this study area (Table 2). The distributions of long-term exposure to PM_2.5_ and NO_2_ among the participants revealed that the IQRs of PM_2.5_ and NO_2_ exposures were 4.1 µg/m^3^ and 4.4 ppb, respectively (Table 2). This demonstrated considerable variations in air pollution exposures across the residential areas of participants. Table 3 presents the Spearman correlation coefficients between the air pollutants. PM_2.5_ was moderately correlated with NO_2_ (r = 0.58) and CO (r = 0.54), and negatively correlated with O_3_ (r = −0.65). NO_2_ also showed a moderate positive correlation with CO (r = 0.62) and a negative correlation with O_3_ (r = −0.58).

**Table 2:** Air pollution exposures during the study period.

	**Mean**	**SD**	**Median**	**Q1**	**Q3**	**Interquartile range**
PM_2.5_, µg/m^3^	23.54	3.65	22.16	20.95	25.04	4.09
NO_2_, ppb	24.79	6.70	23.75	21.54	25.98	4.44
O_3_, ppb	25.62	1.32	25.48	24.76	26.09	1.32
CO, ppm	0.68	0.02	0.68	0.68	0.69	0.01

Definition of abbreviation: PM_2.5_ = particulate matter with a diameter of less than 2.5 micrometers; NO_2_ = nitric oxide; O_3_ = ozone; CO = carbon monoxide.

**Table 3:** Spearman correlation coefficients for air pollution levels during study period.

	**PM_2.5_**	**NO_2_**	**O_3_**	**CO**
PM_2.5_	1	0.58	−0.65	0.54
NO_2_		1	−0.58	0.62
O_3_			1	−0.62
CO				1

Each pairwise Spearman correlation coefficient was statistically significant, with p-values < 0.0001.

In the analysis of annual changes in health outcomes (Table 4), PM_2.5_ showed the largest effect sizes and the most consistent statistical significance across all grip strength and body composition indices, except for SMM of the leg, even after multiple comparison adjustment. NO_2_ and CO were significantly associated with declines in SMM of the arm and trunk, but with smaller effect sizes. In contrast, O_3_ was positively associated with increases in SMM of the arm, trunk, and leg, and inversely associated with BFM of the leg. These findings suggest PM_2.5_ has the strongest adverse impact, while other pollutants may exert more localized or differing effects.

**Table 4:** Single-pollutant models for annual change rate of body composition and grip strength.

**Outcomes**	**Exposures**	**Coefficient (kg/yr)**	**95% CI**	***P* value**	***P*_FDR_**
BFM	PM_2.5_	0.21	(0.13, 0.28)	<0.0001	**<0.0001**
BFM	NO_2_	−0.01	(−0.05, 0.03)	0.609	0.685
BFM	O_3_	−0.04	(−0.1, 0.02)	0.23	0.306
BFM	CO	−0.01	(−0.03, 0.01)	0.407	0.489
SMM	PM_2.5_	−0.14	(−0.18, −0.1)	<0.0001	**<0.0001**
SMM	NO_2_	−0.02	(−0.04, 0.0001)	0.051	0.092
SMM	O_3_	0.05	(0.02, 0.08)	0.003	**0.009**
SMM	CO	−0.01	(−0.02, −0.001)	0.035	0.074
GS	PM_2.5_	−0.16	(−0.27, −0.06)	0.002	**0.007**
GS	NO_2_	−0.04	(−0.1, 0.01)	0.142	0.222
GS	O_3_	0.07	(−0.02, 0.15)	0.125	0.205
GS	CO	−0.03	(−0.06, −0.001)	0.045	0.09
BFM of arm	PM_2.5_	0.03	(0.02, 0.03)	<0.0001	**<0.0001**
BFM of arm	NO_2_	−0.0001	(−0.004, 0.004)	0.959	0.987
BFM of arm	O_3_	−0.01	(−0.01, 0.0002)	0.057	0.097
BFM of arm	CO	−0.001	(−0.003, 0.001)	0.465	0.54
BFM of trunk	PM_2.5_	0.05	(0.01, 0.09)	0.008	**0.021**
BFM of trunk	NO_2_	−0.01	(−0.03, 0.01)	0.392	0.486
BFM of trunk	O_3_	−0.0002	(−0.03, 0.03)	0.99	0.99
BFM of trunk	CO	−0.006	(−0.02, 0.01)	0.351	0.451
BFM of leg	PM_2.5_	0.05	(0.04, 0.07)	<0.0001	**<0.0001**
BFM of leg	NO_2_	−0.0003	(−0.01, 0.01)	0.936	0.987
BFM of leg	O_3_	−0.01	(−0.03, −0.003)	0.01	**0.025**
BFM of leg	CO	−0.001	(−0.005, 0.003)	0.687	0.749
SMM of arm	PM_2.5_	−0.04	(−0.04, −0.03)	<0.0001	**<0.0001**
SMM of arm	NO_2_	−0.006	(−0.01, −0.002)	0.002	**0.006**
SMM of arm	O_3_	0.01	(0.01, 0.02)	<0.0001	**<0.0001**
SMM of arm	CO	−0.002	(−0.004, −0.0004)	0.018	**0.043**
SMM of trunk	PM_2.5_	−0.2	(−0.24, −0.17)	<0.0001	**<0.0001**
SMM of trunk	NO_2_	−0.03	(−0.05, −0.01)	0.003	**0.009**
SMM of trunk	O_3_	0.08	(0.05, 0.11)	<0.0001	**<0.0001**
SMM of trunk	CO	−0.01	(−0.02, −0.002)	0.025	0.056
SMM of leg	PM_2.5_	−0.01	(−0.02, −0.0001)	0.049	0.092
SMM of leg	NO_2_	−0.004	(−0.01, 0.002)	0.17	0.245
SMM of leg	O_3_	0.01	(−0.003, 0.01)	0.201	0.278
SMM of leg	CO	−0.002	(−0.01, 0.001)	0.154	0.231

Abbreviations: BFM, body fat mass; SMM, skeletal muscle mass; GS, grasp strength; *P*_FDR_, False Discovery Rate (FDR)-adjusted p-values

The coefficients were estimated for an IQR increase in exposure to each air pollutants (i.e. 4.1 µg/m^3^ for PM_2.5_, 4.4 ppb for NO_2_, 1.3 ppb for O_3_, and 0.01 ppm for CO).

All models were adjusted for age, sex, body height and weight, education, current smoking, past smoking, hypertension, diabetes mellitus, stroke, heart diseases, asthma, chronic obstructive pulmonary diseases, renal disease, arthritis, physical activity scores, protein intake scores, second-hand smoke exposure, cooking, incense, temperature and relative humidity in 2019, and season of test.

These associations remained robust in two-pollutant models adjusting for NO_2_, O_3_, or CO (Table 5), suggesting the independent effect of PM_2.5_. In contrast, the associations observed for NO_2_, O_3_, and CO in single-pollutant models largely disappeared after mutual adjustment, indicating their effects were confounded by co-pollutants.

**Table 5:** Two-pollutant models for annual change rate of body composition and grip strength.

**Outcomes**	**Exposures**	**with PM_2.5_**	**with NO_2_**	**with O_3_**	**with CO**
BFM	PM_2.5_		0.23**** (0.15,0.31)	0.24**** (0.15,0.33)	0.24**** (0.16,0.32)
SMM	PM_2.5_		−0.14**** (−0.18,−0.1)	−0.14**** (−0.19,−0.1)	−0.14**** (−0.18,−0.1)
GS	PM_2.5_		−0.15** (−0.26,−0.04)	−0.16** (−0.28,−0.04)	−0.14** (−0.25,−0.04)
BFM of arm	PM_2.5_		0.03**** (0.02,0.04)	0.03**** (0.02,0.04)	0.03**** (0.02,0.04)
BFM of trunk	PM_2.5_		0.06** (0.02,0.1)	0.07** (0.02,0.11)	0.06** (0.02,0.1)
BFM of leg	PM_2.5_		0.06**** (0.05,0.07)	0.06**** (0.05,0.07)	0.06**** (0.05,0.07)
SMM of arm	PM_2.5_		−0.04**** (−0.04,−0.03)	−0.04**** (−0.04,−0.03)	−0.04**** (−0.04,−0.03)
SMM of trunk	PM_2.5_		−0.2**** (−0.24,−0.17)	−0.2**** (−0.24,−0.16)	−0.21**** (−0.24,−0.17)
SMM of arm	NO_2_	0.0002 (−0.003,0.004)		−0.003 (−0.01,0.001)	−0.005** (−0.01,−0.001)
SMM of trunk	NO_2_	0.002 (−0.02,0.02)		−0.01 (−0.04,0.01)	−0.03* (−0.05,−0.01)
SMM	O_3_	−0.01 (−0.04,0.03)	0.04* (0.01,0.08)		0.04* (0.005,0.08)
BFM of leg	O_3_	0.01 (−0.003,0.02)	−0.02** (−0.03,−0.005)		−0.02*** (−0.03,−0.01)
SMM of arm	O_3_	−0.0004 (−0.01,0.01)	0.01**** (0.01,0.02)		0.02**** (0.01,0.02)
SMM of trunk	O_3_	−0.002 (−0.03,0.03)	0.07**** (0.04,0.1)		0.09**** (0.05,0.12)
SMM of arm	CO	0.0009 (−0.001,0.003)	−0.002 (−0.004,0.0003)	0.0007 (−0.002,0.003)	

* for p < 0.05, ** for p < 0.01, *** for p < 0.001, **** for p < 0.0001

Abbreviations: BFM, body fat mass; SMM, skeletal muscle mass; GS, grasp strength

The coefficients were estimated for an IQR increase in exposure to each air pollutants (i.e. 4.1 µg/m^3^ for PM_2.5_, 4.4 ppb for NO_2_, 1.3 ppb for O_3_, and 0.01 ppm for CO).

All models were adjusted for age, sex, body height and weight, education, current smoking, past smoking, hypertension, diabetes mellitus, stroke, heart diseases, asthma, chronic obstructive pulmonary diseases, renal disease, arthritis, physical activity scores, protein intake scores, second-hand smoke exposure, cooking, incense, temperature and relative humidity in 2019, season of test, and co-pollutants.

Each IQR increase in long-term PM_2.5_ exposure was significantly associated with an annual decline of 0.16 kg (95% CI: −0.27, −0.06) in grip strength and 0.14 kg (95% CI: −0.18, −0.1) in skeletal muscle mass, as well as a 0.21 kg/year (95% CI: 0.13, 0.28) increase in body fat mass (Table 4). Notably, the estimated effects of PM_2.5_ exposure on annual changes in grip strength and body composition were comparable to, or even greater than, those associated with natural aging in this cohort. For instance, aging was linked to an annual decrease of 0.02 kg in skeletal muscle mass and 0.08 kg in grip strength, as shown in Supplementary Table 2S.

To account for the baseline differences in key outcome variables, such as body fat mass, fat mass of the arm and leg, and skeletal muscle mass of the arm, across PM_2.5_ exposure tertiles, we conducted a sensitivity analysis adjusting for baseline values of each outcome in the regression models. As shown in Supplementary Table 3S, the associations between long-term PM_2.5_ exposure and the annual rates of change in grip strength, skeletal muscle mass, and body fat mass remained robust and statistically significant after these adjustments. These results suggest that the observed associations were not solely driven by baseline differences and reflect independent longitudinal effects of ambient PM_2.5_ exposure on musculoskeletal health in the elderly.

**Fig. 1:**
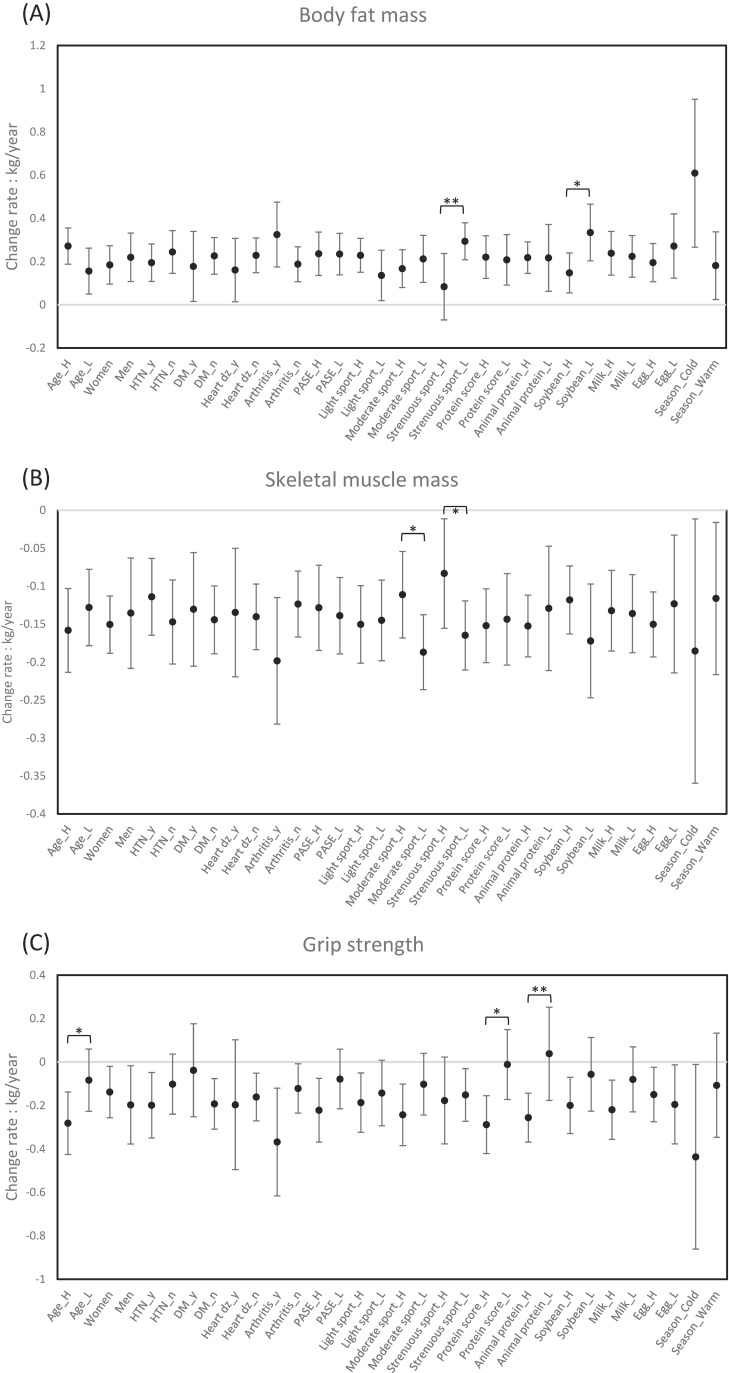
Subgroup analysis of PM_2.5_ exposure and annual change rates in fat mass, muscle, and grip strength. (*p < 0.05, **p < 0.01) The coefficients were estimated for an IQR (4.1 µg/m^3^) increase in exposure to PM_2.5_. All models were adjusted for age, sex, body height and weight, education, current smoking, past smoking, hypertension, diabetes mellitus, stroke, heart diseases, asthma, chronic obstructive pulmonary diseases, renal disease, arthritis, physical activity scores, protein intake scores, second-hand smoke exposure, cooking, incense, temperature and relative humidity in 2019, and season of test.

On the effect modification of personal factors on the observed health impacts of PM_2.5_ (Fig. 1), PM_2.5_ exposures caused a faster decline in grip strength for participants older than 70 years and those having higher protein intake, and in skeletal muscle mass for participants having a lower frequency of moderate or strenuous exercise. PM_2.5_ exposure increased the rate of body fat gain in participants with less strenuous sports or less frequent soybean product intake. In a further subgroup analysis restricted to participants with repeated measurements in both cold and warm seasons across different years, we found no significant differences in the effects of PM_2.5_ on the annual change rates of body fat mass, skeletal muscle mass, or grip strength between seasons, indicating that seasonal variation did not significantly modify these associations.

## Discussion

To our knowledge, this is the first follow-up examining the relationship between air pollutants and markers of sarcopenia, i.e., reduced skeletal muscle and strength, and increased body fat content among the elderly. The observed effects remain consistent even after adjustments for the NO_2_, O_3_, and CO. Participants older than 70 years of age have a higher rate of PM_2.5_-related decline in grip strength. Moderate to strenuous sports or recreational activity provide protective effect for PM_2.5_-related decline in skeletal muscle mass, as well as the increase rate in body fat mass. Higher protein dietary frequency instead increases the rate of PM_2.5_-related decline in grip strength, whereas soybean product intake helps to slow down the rate of PM_2.5_-related body fat gain.

The effects of PM_2.5_ on the annual decline rates of grip strength and skeletal muscle mass are not small compared to the aging effect. In this study, an interquartile range (IQR) increase of 4 µg/m^3^ in PM_2.5_ exposure was associated with a 0.6% annual decline in grip strength, which is approximately twice the rate attributed to aging in our cohort (0.16 kg/year vs. 0.08 kg/year), and corresponds to about 20–30% of the aging-related decline rate (2–4% per year) reported in previous literature [5]. As for muscle mass, the PM_2.5_-related annual decline rate was also 0.6%, which is nearly seven times greater than the aging-related decline observed in our cohort (0.14 kg/year vs. 0.02 kg/year), and is comparable to the aging effect reported in prior studies (0.6–1.0% per year) [5]. The mean concentration of long-term PM_2.5_ exposure in this study is 23.5 µg/m^3^, with an IQR of 4.1 µg/m^3^ (21.0–25.0). This is about the middle level globally [35]. Thus, further studies in areas of higher or lower exposure levels are needed to examine whether a plateau or ceiling effect or a threshold for no observed adverse effect, exists.

In this study, the effect of PM_2.5_ on the annual increase rate in body fat mass is consistent with findings of previous cross-sectional epidemiologic studies showing associations between PM_2.5_ exposure and increased prevalence of obesity [36, 37] and higher body fat mass [38, 39]. Possible mechanisms of PM_2.5_-related body fat gain include effects on impairing adipose tissue anti-oxidative defense [40] and increasing inflammation [41] or affecting sleep [42, 43] and mood [44], which in turn promote metabolic dysfunction and changes in eating behavior. In terms of preventative lifestyles, previous research has shown that exercise can increase antioxidant [45] and anti-inflammatory [46] capacity, reduce body fat [47] and improve sleep [48] and mood [49], supporting our findings on the effects of strenuous exercise or recreational activity on body fat. Furthermore, with regard to soy intake, previous studies have shown that soy protein has anti-obesity, antioxidant and gut microbiota-modifying effects [50, 51], which support our findings and the need for further research.

In single-pollutant models, changes in grip strength and body fat mass were primarily associated with PM_2.5_ exposure, while other pollutants showed limited and non-significant correlations. In contrast, skeletal muscle mass in the arm and trunk was associated with multiple pollutants, suggesting that the effects of air pollution on body composition may be both component- and region-specific. Previous studies have also reported such regional associations, though findings remain inconsistent. For example, a longitudinal cohort study using UK Biobank data found that PM_2.5_ was more strongly associated with increased fat mass in the arm and trunk than in the leg, while NO_2_ showed no significant effect [52]. Another cross-sectional study conducted in Taiwan among individuals with sleep disorders reported negative associations of PM_2.5_ and NO_2_ with fat-free mass in the leg, but positive associations with fat percentage in the arm and leg, respectively [53, 54]. These inconsistencies may reflect differences in study design, air pollution composition, and population characteristics. The underlying mechanisms for region-specific effects, such as the greater susceptibility of arm and trunk muscles, are not yet fully understood. One possible explanation is that leg muscles, due to their weight-bearing role and frequent use, may be more resilient to air pollution-induced damage than muscles in the arm or trunk. Further investigation is warranted to confirm these region-specific effects and to elucidate the biological mechanisms underlying differential muscle susceptibility.

The inverse associations observed between O_3_ and certain body composition outcomes in single-pollutant models should be interpreted cautiously. Similar findings have been reported previously and may reflect confounding rather than true protective effects [53]. In urban settings, high nitric oxide (NO) levels from traffic can reduce ambient O_3_ through titration reactions [55]. In our study, Spearman correlation showed negative associations between O_3_ and other pollutants, suggesting that higher O_3_ levels may indicate lower traffic-related pollution rather than independent health benefits. These results highlight the need for multipollutant models when evaluating air pollution effects.

In this study, even among the elderly, those of older age are more vulnerable to PM_2.5_ compared to the relatively younger participants in terms of decline rate in grip strength. Although the total lung deposition rate of fine particles is comparable between the elderly and adults [56], the clearance rate of inhaled particles in the respiratory tract declines with age, approximately 40–50% lower in the elderly than in young adults [57]. The elderly may therefore retain higher internal dosage of PM_2.5_ than young people. Moreover, anti-oxidative capability decreases with age, which hinders its antagonistic effect on PM_2.5_-induced oxidative stress [3, 58]. Increased accumulation of reactive oxygen species is known to impair muscle fiber activation at neuromuscular junctions, excitation-contraction coupling at ryanodine receptors, and cross-bridging cycling within myofibrillar apparatus, resulting in decreased strength [59]. More efforts to prevent PM_2.5_-induced health hazards on the elderly are needed.

The protective effect of moderate to strenuous sports or recreational activity on skeletal muscle mass is consistent with previous knowledge about the preventive effect of exercise on sarcopenia [60]. Exercise improves muscle mass by increasing protein synthesis, myofibril number, and fiber cross-sectional area. Regular exercise upregulates cellular antioxidant systems and stimulates oxidative damage repair systems [60], which may counter PM_2.5_-induced oxidative stress. In this study, even moderate activity that causes light sweating or increased breathing and heart rate may have beneficial effects on skeletal muscle mass. These findings may increase the feasibility of an effective exercise program for the elderly.

Dietary protein provides amino acids necessary for muscle synthesis. Inadequate protein intake is associated with decreased muscle mass and strength in older adults [61]. Several randomized controlled studies have shown that exercise plus protein supplementation significantly increases muscle mass and function in older adults [62]. However, it is unclear whether or how much additional protein intake is needed in the elderly [62]. Our study showed that a higher frequency of protein diets instead increased the rate of PM_2.5_-related grip strength decline. This result is surprising, but previous research has shown that high-protein diets can increase oxidative stress [63]. While most of the protein ingested (∼40%) is used as an energy source, only about 10% is used for de novo muscle synthesis. Therefore, it is speculated from our findings that additional protein intake does not help counteract the health effects of PM_2.5_ among the elderly.

This study has some limitations. First, there may be unmeasured errors in air pollution exposure assessment. This study only used the residential address to estimate the exposure levels. Since the participants are aged >65 years, a population that is essentially retired, exposure at home may be more relevant. A hybrid Kriging/land-use regression model has been used to estimate long-term exposure to air pollution. The performance of this novel model is good and the cross-validated R-square is as high as 0.88. Thus, the estimation error of air pollution at home is probably small and likely introduces non-differential bias leading to an under-estimation of the observed effects.

Second, there is no measurement of indoor air pollution. Although outdoor PM_2.5_ is the primary source of indoor PM_2.5_, a large number of particles may be generated under certain indoor conditions. However, long-term monitoring of indoor air pollution is currently not feasible in a large epidemiologic study. This study considers several important sources of indoor particulate matter, including second-hand smoke, cooking, and incense. Since this study has been conducted in urban areas, the housing conditions of the participants are very similar. Other uncontrolled indoor PM_2.5_ sources are unlikely related to ambient air pollution and can also be prone to non-differential bias and an under-estimation of health effects.

Third, the study participants have been recruited from those who participate in the hospital’s annual health check, a group that tends to pay more attention to their health. In this study, none of the proportion fulfilled the criteria for diagnosis of sarcopenia at baseline, which indicates that the study participants are relatively healthy elderly. Thus, there should be caution in extending the results to the general elderly population. Further research that includes older people with higher co-morbidities and vulnerability is warranted.

## Conclusion

In conclusion, long-term exposure to ambient PM_2.5_ accelerates declines in grip strength and skeletal muscle mass, and the increase in body fat mass among a relatively healthy elderly population. The magnitude of additional changes related to PM_2.5_ is not trivial. Susceptibility to PM_2.5_ may be influenced by factors such as age, physical activity, and dietary protein intake; however, these modifying effects appear to vary across different health outcomes. Further research is needed to clarify the underlying mechanisms and to assess the consistency of these interactions across populations and settings.

## Abbreviations

PM_2.5_: particulate matter with an aerodynamic diameter of ≤2.5 um
NO_2_: nitrogen dioxide
O_3_: ozone
CO: carbon monoxide
IQR: inter-quartile range
PASE: Physical Activity Scale for the Elderly

## Supplementary information

**Additional file 1:** **Supplementary Table 1S.** Association between PM_2.5_ exposure level groups (low, medium, high) at baseline and during the follow-up period. **Supplementary Table 2S.** Linear mixed-effects model estimates for the association between age and body composition indices and grip strength. Values represent regression coefficients (β) per 1-year increase in age. **Supplementary Table 3S.** Estimated coefficients for the association between air pollution exposure and the annual rate of change in body composition indices and grip strength in single-pollutant models, adjusted for baseline values.

## Declarations

### Availability of data and materials

Data available on request from authors.
