# Supplementary material for: Air pollution exposure associated with decline rates in skeletal muscle mass and grip strength and increase rate in body fat in elderly: a 5-year follow-up study
Source: Environ Health Prev Med. 2025 Jul 18;30:56. doi: 10.1265/ehpm.24-00357 (PMC12301075; doi:10.1265/ehpm.24-00357)
Supplement: Supplementary file 1 — Additional file 1: Supplementary Table 1S. Association between PM2.5 exposure level groups (low, medium, high) at baseline and during the follow-up period. Supplementary Table 2S. Linear mixed-effects model estimates for the association between age and body composition indices and grip strength. Values represent regression coefficients (β) per 1-year increase in age. Supplementary Table 3S. Estimated coefficients for the association between air pollution exposure and the annual rate of change in body composition indices and grip strength in single-pollutant models, adjusted for baseline values. [file ehpm-30-056-s001.docx]

**Supplementary Table 1S.** Association between PM_2.5_ exposure level groups (low, medium, high) at baseline and during the follow-up period

|  | PM_2.5_ during 2015~2019 | | |  |
| --- | --- | --- | --- | --- |
|  | Low | Medium | High | *P* value |
|  | (20.5 μg/m^3^) | (22.2 μg/m^3^) | (27.9 μg/m^3^) |  |
| PM_2.5_ in 2015 |  |  |  | <0.0001 |
| Low (24.3 μg/m^3^) | 125 (94.7) | 7 (5.3) | 0 (0) |  |
| Medium (26.2 μg/m^3^) | 7 (5.3) | 108 (81.8) | 17 (13.0) |  |
| High (31.4 μg/m^3^) | 0 (0) | 17 (13.0) | 114 (87.0) |  |

**Supplementary Table 2S**. Linear mixed-effects model estimates for the association between age and body composition indices and grip strength. Values represent regression coefficients (β) per 1-year increase in age.

| Outcomes (kg) | β(Age) | 95% CI | *P* value |
| --- | --- | --- | --- |
| BFM | -0.09 | (-0.15, -0.03) | 0.002 |
| SMM | -0.02 | (-0.05, 0.01) | 0.129 |
| GS | -0.08 | (-0.16, -0.01) | 0.019 |
| BFM of arm | -0.005 | (-0.01, 0.001) | 0.112 |
| BFM of trunk | -0.03 | (-0.06, -0.003) | 0.031 |
| BFM of leg | -0.02 | (-0.03, -0.01) | <0.0001 |
| SMM of arm | -0.002 | (-0.01, 0.003) | 0.464 |
| SMM of trunk | -0.03 | (-0.05, -0.002) | 0.036 |
| SMM of leg | -0.02 | (-0.03, -0.02) | <0.0001 |

| Abbreviations: BFM, body fat mass; SMM, skeletal muscle mass; GS, grasp strength |
| --- |
| All models were adjusted for age, gender, body height and weight, education, current smoking, past smoking, hypertension, diabetes mellitus, stroke, heart diseases, asthma, chronic obstructive pulmonary diseases, renal disease, arthritis, physical activity scores, protein intake scores, second-hand smoke exposure, cooking, incense, temperature, relative humidity, and season of test. |

**Supplementary Table 3S.** Estimated coefficients for the association between air pollution exposure and the annual rate of change in body composition indices and grip strength in single-pollutant models, adjusted for baseline values.

| Outcomes | Exposures | Coefficient (kg/yr) | 95% CI | *P* value | *P*_FDR_ | |
| --- | --- | --- | --- | --- | --- | --- |
| BFM | PM_2.5_ | 0.21 | (0.13, 0.28) | <0.0001 | <0.0001 | |
| BFM | NO_2_ | -0.01 | (-0.06, 0.03) | 0.539 | 0.607 | |
| BFM | O_3_ | -0.04 | (-0.1, 0.02) | 0.229 | 0.317 | |
| BFM | CO | -0.01 | (-0.03, 0.01) | 0.41 | 0.492 | |
| SMM | PM_2.5_ | -0.14 | (-0.18, -0.1) | <0.0001 | <0.0001 | |
| SMM | NO_2_ | -0.02 | (-0.04, -0.0002) | 0.048 | 0.093 | |
| SMM | O_3_ | 0.05 | (0.02, 0.08) | 0.004 | 0.011 | |
| SMM | CO | -0.01 | (-0.02, -0.001) | 0.034 | 0.072 | |
| GS | PM_2.5_ | -0.16 | (-0.27, -0.06) | 0.002 | 0.006 | |
| GS | NO_2_ | -0.04 | (-0.1, 0.02) | 0.155 | 0.235 | |
| GS | O_3_ | 0.06 | (-0.02, 0.15) | 0.144 | 0.235 | |
| GS | CO | -0.03 | (-0.06, -0.0001) | 0.049 | 0.093 | |
| BFM of arm | PM_2.5_ | 0.03 | (0.02, 0.03) | <0.0001 | <0.0001 | |
| BFM of arm | NO_2_ | -0.0003 | (-0.005, 0.004) | 0.879 | 0.916 | |
| BFM of arm | O_3_ | -0.01 | (-0.01, 0.0003) | 0.061 | 0.105 | |
| BFM of arm | CO | -0.001 | (-0.003, 0.001) | 0.442 | 0.514 | |
| BFM of trunk | PM_2.5_ | 0.05 | (0.01, 0.09) | 0.007 | 0.02 | |
| BFM of trunk | NO_2_ | -0.01 | (-0.03, 0.01) | 0.326 | 0.419 | |
| BFM of trunk | O_3_ | -0.0003 | (-0.03, 0.03) | 0.985 | 0.985 | |
| BFM of trunk | CO | -0.01 | (-0.02, 0.01) | 0.36 | 0.447 | |
| BFM of leg | PM_2.5_ | 0.05 | (0.04, 0.07) | <0.0001 | <0.0001 | |
| BFM of leg | NO_2_ | -0.0005 | (-0.01, 0.01) | 0.891 | 0.916 | |
| BFM of leg | O_3_ | -0.01 | (-0.02, -0.004) | 0.009 | 0.024 | |
| BFM of leg | CO | -0.001 | (-0.005, 0.003) | 0.688 | 0.751 | |
| SMM of arm | PM_2.5_ | -0.04 | (-0.04, -0.03) | <0.0001 | <0.0001 | |
| SMM of arm | NO_2_ | -0.01 | (-0.01, -0.002) | 0.001 | 0.006 | |
| SMM of arm | O_3_ | 0.01 | (0.01, 0.02) | <0.0001 | <0.0001 | |
| SMM of arm | CO | -0.002 | (-0.004, -0.0003) | 0.021 | 0.051 | |
| SMM of trunk | PM_2.5_ | -0.2 | (-0.24, -0.17) | <0.0001 | <0.0001 | |
| SMM of trunk | NO_2_ | -0.03 | (-0.05, -0.01) | 0.003 | 0.008 | |
| SMM of trunk | O_3_ | 0.08 | (0.05, 0.11) | <0.0001 | <0.0001 | |
| SMM of trunk | CO | -0.01 | (-0.02, -0.001) | 0.028 | 0.063 | |
| SMM of leg | PM_2.5_ | -0.01 | (-0.02, 0.0004) | 0.06 | 0.105 | |
| SMM of leg | NO_2_ | -0.004 | (-0.01, 0.002) | 0.176 | 0.254 | |
| SMM of leg | O_3_ | 0.01 | (-0.004, 0.01) | 0.245 | 0.327 | |
| SMM of leg | CO | -0.002 | (-0.01, 0.001) | 0.157 | 0.235 | |
| Abbreviations: BFM, body fat mass; SMM, skeletal muscle mass; GS, grasp strength; *P*_FDR_, False Discovery Rate(FDR)-adjusted p-values | | | | | |  |
| The coefficients were estimated for an IQR increase in exposure to each air pollutants (i.e. 4.1 μg /m^3^ for PM_2.5_, 4.4 ppb for NO_2_, 1.3 ppb for O_3_, and 0.01 ppm for CO). | | | | | |  |
| All models were adjusted for age, gender, body height and weight, **baseline indices of body composition or grip strength**, education, current smoking, past smoking, hypertension, diabetes mellitus, stroke, heart diseases, asthma, chronic obstructive pulmonary diseases, renal disease, arthritis, physical activity scores, protein intake scores, second-hand smoke exposure, cooking, incense, temperature, relative humidity, season of test, and co-pollutants. | | | | | |  |
